# A novel tropoelastin-based resorbable surgical mesh for pelvic organ prolapse repair

**DOI:** 10.1016/j.mtbio.2020.100081

**Published:** 2020-10-13

**Authors:** B. Aghaei-Ghareh-Bolagh, S. Mukherjee, K.M. Lockley, S.M. Mithieux, Z. Wang, S. Emmerson, S. Darzi, C.E. Gargett, A.S. Weiss

**Affiliations:** a Charles Perkins Centre, University of Sydney, NSW, 2006, Australia; b School of Life and Environmental Sciences, University of Sydney, NSW, 2006, Australia; c The Ritchie Centre, Hudson Institute of Medical Research, Victoria, 3168, Australia; d Department of Obstetrics and Gynaecology, Monash University, Victoria, 3168, Australia; e The University of Sydney Nano Institute, University of Sydney, NSW, 2006, Australia

**Keywords:** Elastin, Tropoelastin, Pelvic organ prolapse, Surgical mesh, Degradable, Vaginal repair

## Abstract

Pelvic organ prolapse is a common condition that affects 1 in 4 women across all age groups. It is mainly caused by vaginal birth injury and can be exacerbated by obesity and increased age. Until recently, treatment strategies often used non-degradable synthetic meshes for reconstructive surgery. However, owing to their frequent, unacceptable rate of adverse events such as mesh erosion, transvaginal meshes have been banned in many countries. Recent reports have highlighted the urgent need for biocompatible design of meshes for a safe and effective treatment in the long term.

This study reports the design and evaluation of a novel, elastin based degradable mesh using an ovine model of POP as a potential surgical treatment. Elastin is a protein component of the ECM and provides elasticity to tissues throughout the body. Tropoelastin, the monomer subunit of elastin, has been used with success in electrospun constructs as it is a naturally cell interactive polymer. Biomaterials that incorporate tropoelastin support cell attachment and proliferation, and have been proven to encourage elastogenesis and angiogenesis *in vitro* and *in vivo*.

The biological properties of tropoelastin were combined with the physical properties of PCL, a degradable synthetic polymer, with the aim of producing, characterizing and assessing the performance of continuous tropoelastin:PCL electrospun yarns. Using a modified spinneret electrospinning system and adjusting settings based on relative humidity, four blends of tropoelastin:PCL yarns were fabricated with concentration ratios of 75:25, 50:50, 25:75 and 0:100. Yarns were assessed for ease of manufacture, fibrous architecture, protein/polymer content, yarn stability - including initial tropoelastin release, mechanical strength, and ability to support cell growth. Based on overall favorable properties, a mesh woven from the 50:50 tropoelastin:PCL yarn was implanted into the vagina of a parous ewe with vaginal wall weakness as a model of pelvic organ prolapse. This mesh showed excellent integration with new collagen deposition by SEM and a predominant M2 macrophage response with few pro-inflammatory M1 macrophages after 30 days. The woven tropoelastin:PCL electrospun mesh shows potential as an alternative to non-degradable, synthetic pelvic organ prolapse mesh products.

## Graphical abstract

**Figure:**
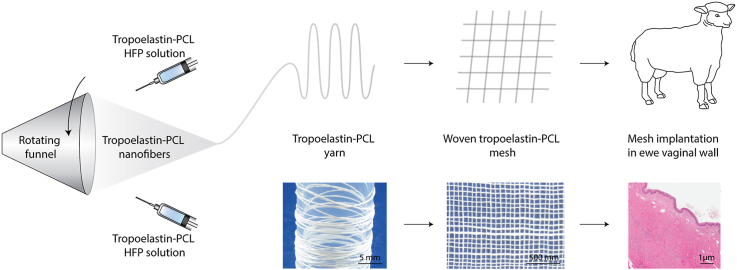

## 1 Introduction

Pelvic organ prolapse (POP) is a debilitating condition that affects 25% of all women [1]. POP occurs when the pelvic support structures; suspensory ligaments, vaginal wall and pelvic floor muscles are damaged from vaginal birth and weaken over time, causing the downward descent of pelvic organs [2,3]. Symptoms include bladder, bowel and sexual dysfunction, feeling of a bulge in the vagina and less commonly urinary and fecal incontinence. Risk factors for POP include childbirth, obesity and increased age [1]. Non-degradable synthetic meshes have been used for decades for abdominal hernia repair and more recently for the surgical repair of vaginal tissue in women with POP, however their use is now severely restricted due to company withdrawal of vaginal mesh and regulatory authority bans on their use in many countries including the USA, UK, Australia and New Zealand. Reported complications leading to these bans were mesh erosion into pelvic organs, mesh exposure, infections and pain requiring further surgeries for their removal [4,5]. The 20% of women requiring POP reconstructive surgery are now faced with limited treatment options as native tissue surgery fails in ~30% of cases [6,7].

Recent reports have highlighted that implantation of non-degradable polypropylene meshes lead to significant disruption of vaginal extracellular matrix (ECM) [8]. Furthermore, such meshes are also associated with an undesirable immune response, which together with biomechanical mismatch between mesh and vaginal tissue, are responsible for mesh-related complications in women such as erosion/exposure and chronic pain [9]. In order to overcome the current limitations and complications of non-degradable meshes, recent research has focused on developing meshes using degradable polymers, particularly using electrospinning that promotes cellular attachment, proliferation and formation of new ECM [10,11]. Recently, several groups have shown that electrospun constructs fabricated with degradable polymers have significant potential in POP treatment using small and large animal models [[12], [13], [14], [15], [16]]. We have previously shown that choice of polymer and surface topography largely influences the triggered *in vivo* immune response [17]. These features, and physical and mechanical properties such as fibrous architecture and large pore size (one mm diam), determine cellular attachment and infiltration, cytokine release and ectopic recruitment of tissue forming cells. From a clinical perspective, meshes for POP treatment that allow for infiltration of cells, growth of new tissue and promote an anti-inflammatory response will reduce adverse events such as mesh erosion, and the need for surgical removal can be avoided [18]. Furthermore, the materials must be non-toxic, and any by-products produced during the breakdown should not interfere with or harm the surrounding tissue at implant site [10,19]. Previously, meshes for POP application were mechanically too stiff compared to native tissue. It is now known that stiff and non-elastic POP meshes contribute to mesh erosions and exposures and an undesired immune response [14,20]. Vaginal tissue is viscoelastic and can endure significant stretching during childbirth. New generation POP meshes need to be lightweight and possess mechanical properties that closely match the physical requirements of tissue at the implantation site [21,22]. An ideal mesh for vaginal application would be an elastic and biocompatible mesh that provides enough strength to support the pelvic organs and ultimately integrates into the native tissue.

Electrospinning is a versatile technique used to fabricate fibers that has recently gained attention in urogynecological applications [15,17,23]. Electrospinning provides an adaptable path for the construction of nanofibers and microfibers with defined fiber morphology, dimensions and orientation, which present the multiple benefits of a high surface-to-volume ratio and twisting to generate yarns, which is conducive to delivering reproducible textiles [24] as demonstrated here. The process is scalable as evidenced by commercial electrospinning companies that provide contract manufacturing [25]. To improve the surgical applicability of electrospun biomaterials, the traditional method can be modified to derive a continuous yarn [26] comprising aligned fibers that form a twist, which increases tensile strength and flexibility of the yarn. These continuous yarns are capable of being woven into more complex structures and possess the ability to withstand mechanical stress as needed to support load-bearing tissues in the body [26,27], an important consideration for vaginal repair.

Elastin is one of the connective tissue components that make up the extracellular matrix (ECM) and is found throughout the body in elastic tissues such as skin and blood vessels [28], where it provides resilience to these tissues so they can withstand continuous strain [29]. Elastin is cell interactive and influences cellular attachment [30,31], proliferation [28] and differentiation [32]. Tropoelastin is the predominant precursor of elastin, and interacts with a range of cells to promote wound healing through a combination of angiogenesis, chemotaxis, proliferation, differentiation and elastic extracellular matrix synthesis [33]. Tropoelastin electrospun fibers have been proven to support cell growth and promote proliferation [34] and are also well tolerated *in vivo* [35,36].

Polycaprolactone (PCL) is a synthetic, non-toxic degradable polymer [37,38] that has been approved for use in certain biomedical applications by the US Food and Drug Administration [18]. PCL has been used to make electrospun fibers that have a low *in vivo* degradation rate [39] and have been used successfully in dermal [38] and tendon repair [22]. Blending of PCL-based fibers with natural polymers significantly improves their biological compatibility as well as *in vivo* performance [13,38,40,41].

Here, we report that the biological and physical properties of tropoelastin, combined with the favorable physical properties of PCL, generate hybrid electrospun fibrous yarns in multimeter lengths that are degradable and capable of supporting cellular growth. We further show, for the first time, the potential of these hybrid yarns in woven vaginal mesh for tissue engineering applications in an ovine model of POP.

## 2 Materials and methods

### 2.1 Electrospinning

Recombinant human tropoelastin (corresponding to amino acid residues 27–724 of GenBank entry AAC98394 (https://www.ncbi.nlm.nih.gov/protein/AAC98394) isoform SHELΔ26A) was obtained from Elastagen. Four blends of tropoelastin and PCL (Mw = 80,000 g/mol; Sigma-Aldrich) were prepared using 10% (w/v) solutions of tropoelastin and PCL dissolved separately in hexafluoroisopropanol (HFP, Sigma-Aldrich). After dissolution for 18 h at 4 °C the 10% (w/v) tropoelastin and 10% (w/v) PCL solutions were blended together for 4 h on a rotating platform (Ratek, Australia) in the following tropoelastin:PCL ratios – 75:25, 50:50, 25:75, and 0:100.

A purpose-built modified dual spinneret electrospinning system based on Ali et al. [26], combination with parameters that we had defined previously for the fabrication of tropoelastin:silk hybrid yarns [24], were used in this study. The electrospinning system was housed inside a poly (methyl methacrylate) (PMMA) box and the temperature was kept constant at 20 °C. Humidity was measured using an AcuRite indoor humidity monitor placed inside the box. In this system, two 1 mL syringes attached to 18-gauge needles were loaded with a tropoelastin:PCL blend and positioned facing a rotating funnel collector. One needle was connected to a 10 kV positive power supply and the other to a 10 kV negative power supply to generate an electric field. Syringe pumps were used to pass the tropoelastin:PCL blends through the needle connected to the positive power supply at a constant rate of 1.8 mL/min, and through the needle connected to the negative power supply at a constant rate of 1.2 mL/min. As the charged polymer solutions moved towards the rotating funnel collector (10 cm diameter), the HFP evaporated and a flat fibrous mat formed across the collector. A plastic rod was then used to shape a fibrous cone from which a fibrous yarn could be drawn and collected around a rotating winder (2 cm diameter).

### 2.2 Structural characterization

Scanning electron microscopy (SEM) was used to characterize the tropoelastin:PCL electrospun yarns. Yarns were mounted with silver conductive paint and then sputter-coated with 15 nm gold. SEM images were collected for measurements using a JEOL Neoscope Tabletop SEM (JEOL, Japan). Image J software (version 1.52a, National Institutes of Health, USA) was used to measure yarn diameter and nanofiber angle. To determine fiber diameter, Image J software was used to draw a line across each yarn and the widths of the first ten fibers were measured for each yarn blend (n = 3).

The effect of immersion in an aqueous solution on fiber structure was analyzed using a high-resolution Zeiss Sigma HD field emission gun scanning electron microscopy (FEG SEM) (Zeiss, France). Yarns were immersed in Milli-Q water (MQW) at 37 °C for 24 h, rinsed 3x with MQW then dried overnight at 37 °C. Prior to imaging, yarns were mounted with silver conductive paint and then sputter coated with 15 nm gold.

### 2.3 Characterization of chemical composition

The chemical compositions of the four tropoelastin:PCL yarn blends were assessed by Fourier transform infrared spectroscopy (FTIR) performed on a Bruker LUMOS FTIR Microscope spectrometer (Bruker, USA) fitted with a micro-ATR pressure-controlled crystal. For each measurement, 64 scans were averaged with a 4 cm^−1^ resolution at medium pressure. Spectral analysis was performed using OPUS software version 7.5 (Cooperative Library Network Berlin-Brandenburg, Germany). Atmospheric compensation and baseline correction were applied to all spectra.

### 2.4 Stability

Yarn stability and tropoelastin-release in Phosphate Buffered Saline (PBS) was investigated.

For each blend, 2 mg yarn was treated with 1 mL absolute ethanol for 10 min. Samples were left in a biosafety hood for 1 h to allow ethanol to evaporate, before each yarn was immersed in 1 mL sterile PBS under aseptic conditions and placed at 37 °C for 7 days. At days 1, 3, 5 and 7, 70 μL PBS was removed from each sample and frozen at −80 °C, then replaced with 70 μL fresh PBS. Protein release was qualitatively assessed using sodium dodecyl sulphate-polyacrylamide gel electrophoresis (SDS-PAGE) to assess if tropoelastin was released from the tropoelastin:PCL yarns. Loading buffer (1.5 μL) (Life Technologies, USA) was added to the PBS samples (4.5 μL). The samples were then heat denatured at 95 °C for 5 min. Samples and Mark12 unstained protein standard (Life Technologies, USA) were loaded onto a 4–12% NuPAGE Bis-Tris gel (Life Technologies, USA) and run at 200 V for 35 min in NuPAGE MES SDS running buffer (Life Technologies, USA). The gel was then fixed with 50% (v/v) methanol for 30 min and then stained with Coomassie stain solution for 1 h. The gel was de-stained with 25% (v/v) methanol and 10% (v/v) acetic acid for 1 h. For quantitative analysis of tropoelastin release, a NanoDrop 2000c UV–visible spectrophotometer (Thermo Fisher Scientific, USA) was used to measure protein concentration in the PBS samples. Two μL of each sample was loaded onto the pedestal and absorbance was measured at 280 nm in triplicate.

The effect of ethylene oxide sterilization on tropoelastin release from 50:50 tropoelastin:PCL meshes was also assessed by sodium dodecyl sulfate-polyacrylamide gel electrophoresis (SDS-PAGE) analysis. Two tropoelastin:PCL (50:50) meshes (1 × 1 cm, pore size of 1 mm^2^) with and without ethylene oxide sterilization with dry weights of 1.1 mg and 1.8 mg respectively were placed in each well of a 24-well plate. The ethylene oxide sterilized mesh was incubated in 0.5 mL PBS and the second mesh was incubated in 100% ethanol. After 30 min the solutions were removed and replaced by 0.5 mL PBS. Replacement with further PBS was repeated at 1 h, 3 h and 18 h. Samples were then run on an SDS-PAGE gel alongside 5 μg tropoelastin for comparison.

### 2.5 Mechanical testing

Mechanical testing was performed as described [24] on 50:50 tropoelastin:PCL yarns (3.5 cm) and 50:50 woven meshes (1 × 3.5 cm) that were secured in rectangular shaped aluminum foil frames. Yarns and meshes were tested after 20 h incubation in PBS at 37 °C. All measurements were performed on samples suspended in a PBS bath heated to 37 °C and collected using an Instron 5560 tensile testing machine with a 100 N capacity load cell. The yarns were stretched at a constant strain rate of 100 mm/min until break while the meshes were first repeatedly stretched to 15% strain at a rate of 50 mm/min for 10 cycles and then stretched to break at a strain rate of 100 mm/min.

### 2.6 Cell culture

Human dermal fibroblasts (GM3348, Coriell Institute, USA) were cultured in Dulbecco's Modified Eagle's Medium (DMEM, Life Technologies, USA), supplemented with 10% Fetal Bovine Serum (FBS, Life Technologies, USA) and 1% penicillin-streptomycin (Life Technologies, USA) at 37 °C and 5% CO_2_. Fibroblast growth was assessed on yarns and meshes produced from 50:50 blends of tropoelastin:PCL. Samples were mounted into 24-well plate Cell Crown inserts (Sigma-Aldrich, USA), immersed in absolute ethanol (Ajax Finechem, Australia) for 10 min then dried sterile, and seeded with 2.5 × 10^4^ fibroblasts/well and cultured for 7 days. Cell Crown inserts were used to keep the samples flat during cell seeding and to assist in the transfer of samples to fresh wells for cell proliferation analysis. Cell culture media were aspirated and replaced with fresh culture media after 24 h, and then every 48 h.

Cell growth on the yarns was assessed microscopically. Seven days after seeding, yarns were washed 3x with PBS, fixed with 10% formalin (Sigma-Aldrich, USA) for 24 h at room temperature and washed 3× with PBS. The yarns were placed in 0.2% Triton X-100 (Sigma-Aldrich, USA) for 6 min and rinsed 3x with PBS. Cells on the yarns were then incubated for 30 min in the dark with ActinRed 555 ReadyProbes (Thermo Fisher Scientific, USA) to stain F-actin, and TO-PRO 3 iodide (Thermo Fisher Scientific, USA) to stain nuclei. Yarns were washed 3× with PBS and then confocal images were collected using a Nikon Ti-E Spinning Disk microscope (Nikon, Japan). Excitation/emission wavelengths were 540/565 nm and 642/661 nm for ActinRed 555 ReadyProbes and TO-PRO 3 iodide staining, respectively.

For proliferation studies on the 50:50 mesh (n = 3), the DNA content of cultured cells at days 1, 4 and 7 was measured using CyQuant (Invitrogen) cell proliferation assay kits [24].

### 2.7 Fabrication of 50:50 tropoelastin:PCL mesh for *in vivo* study

For each mesh, a continuous yarn of 50:50 tropoelastin:PCL was woven with a spaced, plain weave using an evenly distributed warp and weft with mean pore sizes of 1.0 mm, then formed as rectangular (2 × 3 cm) mesh. Sterilization by ethylene oxide was performed according to ISO 11135:2014 prior to surgery.

### 2.8 Surgical implantation of 50:50 tropoelastin:PCL mesh into ovine vagina

Experimental procedures and animal husbandry were approved by the Monash Medical Centre Animal Ethics Committee A in accordance with the ethical guidelines of the National Health and Medical Research Council (NHMRC) of Australian Code for the Care and Use of Animals for Scientific Purposes.

Multiparous Border Leicester Merino (BLM) ewes which had delivered lambs at least 3 times were chosen after a vaginal examination using our modified POP-quantification based on the human POP-Q system [[42], [43], [44]]. Vaginal surgeries were performed in two sheep: (a) tropoelastin:PCL (50:50) mesh and (b) an incision control using our established method [43]. Anesthesia was induced by intravenous medetomidine medication instituted beforehand (0.1–0.2 mg/kg) followed by intravenous thiopentone (10 mg/kg), and then maintained with isoflurane (1–3% in 100% O_2_). Pain relief was provided before start of surgery as fentanyl (75 μg/h) transdermal patch and subcutaneous carprofen (2 mg/kg). A short acting broad-spectrum antibiotic, cefazolin (7.5 mg/kg), was given intravenously prior to surgery, and a long-acting antibiotic, duplocilin (5.75 mg/kg), to continue coverage for 48 h after surgery. Ewes were placed into a lithotomy position followed by hydrodissection of the vaginal tissue layers with 20 mL bupivacaine (5 mg/mL) and 1 mL adrenaline (Aspen Pharmacare Australia, 1 mg/mL). A 40 mm full-thickness midline incision was made on the posterior vaginal wall and the rectovaginal space was dissected. 2 × 3 cm 50:50 tropoelastin:PCL mesh was surgically implanted and fixed with absorbable sutures into the vaginal wall, and the vaginal epithelium closed using absorbable sutures. Additional pain relief was bupivicaine (5 mg/mL) given subcutaneously at the incision site at end of surgery.

### 2.9 Histological analysis of ovine vaginal tissue

Ewes were euthanized after 30 days using lethabarb (110 mg/kg, Virbac, Australia), and the whole vaginal tract was explanted, trimmed and tissue areas with mesh were identified, dissected and fixed using 10% formalin and 4% paraformaldehyde and embedded into paraffin and frozen blocks, respectively.

Paraffin blocks were sectioned at 8 μm and stained with hematoxylin & eosin (H&E), Gomori Trichome, Picrosirius Red and Verhoeff-van Gieson (VVG) collagen and elastin stains in the Monash Histology Platform (MHP) using previously published methods. Images were obtained by Aperio scanning or using an Olympus BX61 light microscope. Sirius Red stained slides were observed using light microscope Olympus DP80 camera and CellSens software. Using polarizing filters, birefringent collagen fibers were separated into green and red representing immature to mature collagen fibrils, respectively [43].

Immunohistochemistry staining was performed on FFPE sections following antigen retrieval using 0.1 M citrate buffer, blocking endogenous peroxidase with 3% H_2_O_2_, incubation with protein block (Dako) for 30 min at room temperature, using mouse anti-CD45 (0.5 μg/mL, BioRad) and mouse anti-CD206 (0.5 μg/mL, Dendritics) primary antibodies for 1 h at 37C. Isotype matched IgG antibodies at the same concentration were used as negative controls. HRP-labelled polymer (Dako) conjugated anti-mouse secondary antibody was applied for 40 min at room temperature and DAB chromogen (Sigma-Aldrich).

Immunofluorescence staining was performed on paraformaldehyde (PFA)-fixed cryosections blocked with protein block using mouse anti-CD45 and rat anti-CD206 and incubated for 1 h at room temperature. Anti-mouse conjugated Alexa-488 and anti-rat conjugated Alexa 568 secondary antibodies (both Thermo-Fisher) were then incubated for 30 min at room temperature. Nuclei were stained with Hoechst 33258 (Molecular Probes) for 5 min. Collagen III immunofluorescence antigen retrieval was with 0.1% Triton X for 90 s, then a protein block was applied followed by rabbit anti-collagen III alpha 1 (1/50, Novus) for 1 h at room temperature and Alexa-488 anti-rabbit secondary antibody and Hoechst 33258. Images were captured using a FV1200 confocal microscope.

### 2.10 Statistical analysis

Data were expressed as mean ± standard deviation and analyzed using one-way or two-way analysis of variance (ANOVA) using GraphPad Prism version 7.0b software (GraphPad Software, USA). Tukey's multiple comparison test was used to determine significant difference between different conditions. Data for *in vitro* studies were statistically significant when *p* < 0.05. Significant difference is indicated in the figures as ∗ = p < 0.05, ∗∗ = *p* < 0.01, ∗∗∗ = *p* < 0.001. ns = no significance.

## 3 Results and discussion

### 3.1 Electrospinning

**Fig. S1:**
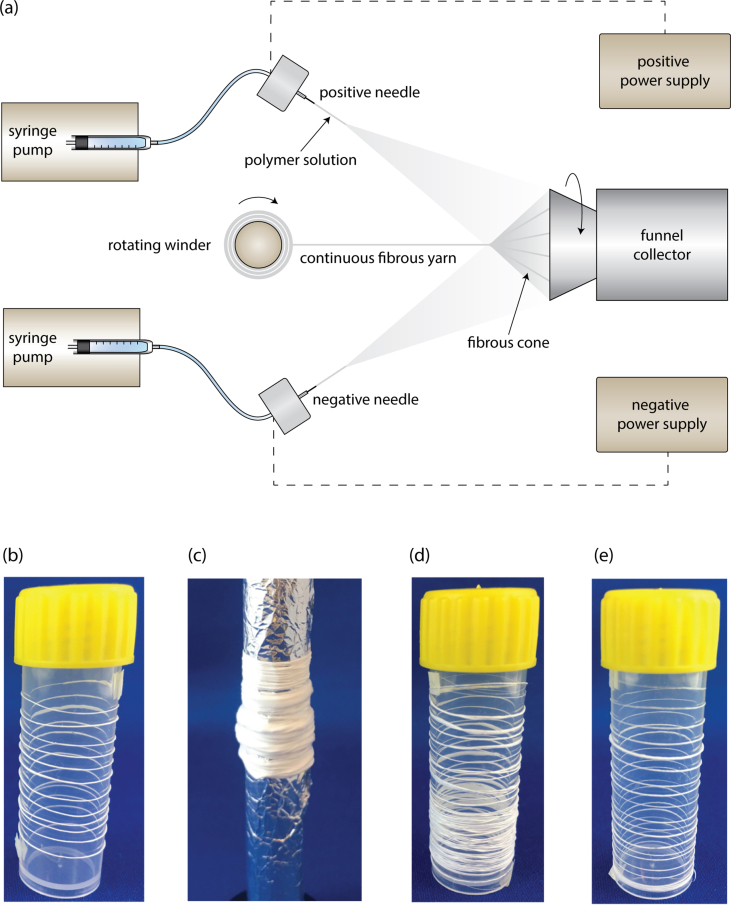
(a) Schematic showing electrospinning setup to generate spun yarns. Photographs of tropoelastin:PCL electrospun yarns produced using optimized electrospinning parameters resulting in (b) 75:25, (c) 50:50 (d), 25:75, and (e) 0:100 electrospun yarn.

A dedicated electrospinning system (ES) [26] was used to fabricate continuous yarns consisting of four different blends of the natural protein tropoelastin and the synthetic polymer polycaprolactone (PCL). A schematic representation of the electrospinning setup is shown in Supp. Fig. 1a. Fig. S1 Tropoelastin and PCL solutions (10% w/v) were mixed in tropoelastin:PCL ratios of 75:25, 50:50, 25:75 and 0:100, where the 0:100 ratio was equivalent to 100% PCL. Hexafluoroisopropanol (HFP) was used for fabrication of the yarns as it has been used to electrospin tropoelastin fibers that support cell growth *in vitro* and were well tolerated in *in vivo* studies [45,46]. Electrospinning was at constant temperature, however relative humidity levels varied, which necessitated adjustments to the funnel speed and winder speed (each stated in revolutions per minute) in order to produce continuous tropoelastin:PCL yarns. Yarns were produced using a prototype assembly, and future studies will focus on use of a dedicated setup to improve the reproducibility of yarn length. Representative images of the electrospun yarns are shown in Supp. Fig. 1b–e and the average yarn length and electrospinning parameters for each blend are noted in Table 1. The longest yarns of 408 ± 234 cm were produced with 50:50 tropoelastin:PCL. The ability to fabricate homogeneous yarns of multimeter lengths facilitated their weaving into more complex structures, including mesh constructs for surgical use [22].

**Fig. 1:**
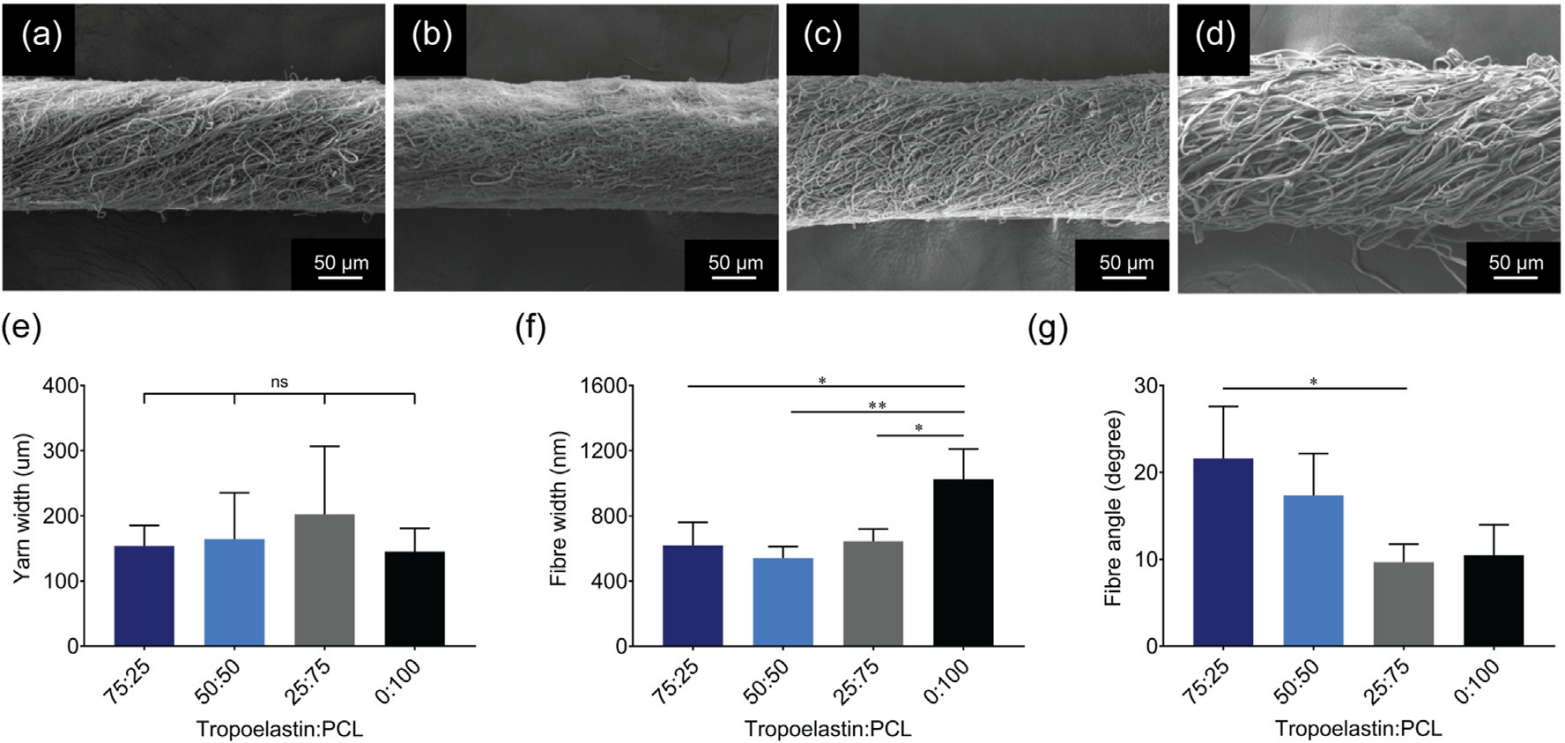
Yarn structure. SEM micrographs of (a) 75:25, (b) 50:50, (c) 25:75 and (d) 0:100 tropoelastin:PCL electrospun yarns. Measurements of (e) yarn width, (f) fiber width, (g) fiber angle. Data show the mean ± standard deviation. For each group n = 3.

**Table 1:** Average tropoelastin:PCL yarn lengths using adjusted funnel collector speed and rotating winder speed.

Tropoelastin:PCL	Average yarn length (cm) (n = 3)	Relative humidity (%)	Funnel collector speed (RPM)	Rotating winder speed (RPM)
75:25	91 ± 25	40–55	750–1000	10
50:50	408 ± 234	45–56	875–1000	10
25:75	253 ± 61	36–48	750–1000	8–10
0:100	113 ± 27	52–55	1000	10

### 3.2 Structural characterization

Scanning electron microscopy (SEM) was used to assess the fibrous architecture of the four tropoelastin:PCL yarn blends (Fig. 1a–d) and to determine yarn width, fiber width and fiber angle. Yarn widths (Fig. 1e) were similar for all four blends with average measurements of 153 ± 32 μm for 75:25, 165 ± 71 μm for 50:50, 202 ± 105 μm for 25:75 and 145 ± 36 μm for 0:100 yarns. All yarns comprised aligned fibers that were angled to produce a twist in the yarn. Average fiber widths within each yarn blend are shown in Fig. 1f. Fibers within the 0:100 yarns were 1025 ± 186 nm in width which was similar to those reported for electrospun PCL [47,48]. The incorporation of tropoelastin into yarns significantly decreased their fiber widths to nanofiber thicknesses. The 75:25, 50:50 and 25:75 tropoelastin:PCL yarns contained fibers that were 618 ± 144 nm, 541 ± 70 nm and 645 ± 75 nm wide, respectively. Larger twist angles contribute to increased flexibility and tensile strength [26]. Average fiber twist angles (Fig. 1g) for the 75:25, 50:50, 25:75 and 0:100 tropoelastin:PCL yarns were 22 ± 6°, 17 ± 5°, 10 ± 2° and 10 ± 3° respectively, such that decreasing amounts of tropoelastin gave rise to smaller twist angles.

### 3.3 Characterization of chemical composition

**Fig. 2:**
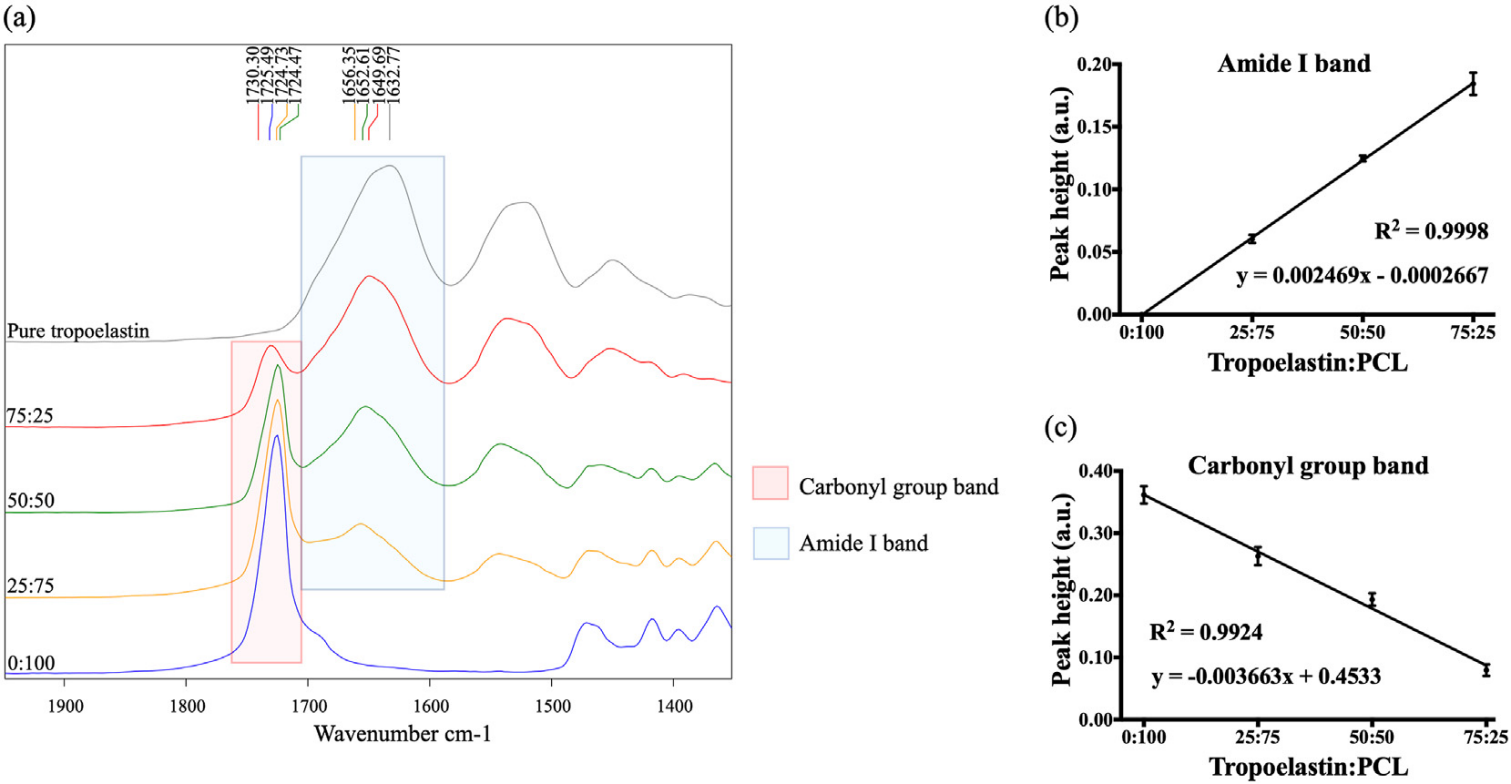
Yarn composition. (a) Comparison FTIR-ATR offset spectra of tropoelastin:PCL electrospun yarns and pure tropoelastin. Representative FTIR-ATR spectra from 1950 to 1350 cm^−1^. (b) FTIR-ATR spectral peak height of Amide I band of tropoelastin:PCL electrospun yarns. (c) Spectral peak height of carbonyl group band of tropoelastin:PCL electrospun yarns. Data show the mean ± standard deviation, for each group n = 3.

Fourier transform infrared spectrometry–attenuated total reflectance (FTIR-ATR) analysis was used to characterize the surface protein/polymer composition of each blend of electrospun tropoelastin:PCL yarn. FTIR-ATR spectra revealed changes commensurate with the ratios of tropoelastin:PCL (Fig. 2a). The carbonyl group band (~1724–1730 cm^−1^) was used to measure the polymer component as this is attributed to the stretching vibration of the C=O bond in PCL [48]. This band was absent from the pure tropoelastin spectra. The peak height of the carbonyl group band decreased with lower proportions of PCL. The amide I band (~1632–1656 cm^−1^) was used to measure the protein component [49]. This band was absent in 0:100 tropoelastin:PCL spectra, consistent with the absence of tropoelastin in this blend. The amide I band peak height also decreased as the proportion of tropoelastin in each yarn decreased. The relationship between peak height and concentration of tropoelastin and PCL in each tropoelastin:PCL blend is shown in Fig. 2b and c respectively. The high correlation of the amide I band and carbonyl group band (R^2^ = 0.9998 and R^2^ = 0.9924 respectively) was consistent with a model where variations in the amide I band and carbonyl group band peak heights were due to the amounts of tropoelastin and PCL in polymer mixtures prior to electrospinning, and thus confirmed the amounts of tropoelastin and PCL in each yarn.

### 3.4 Stability

**Fig. 3:**
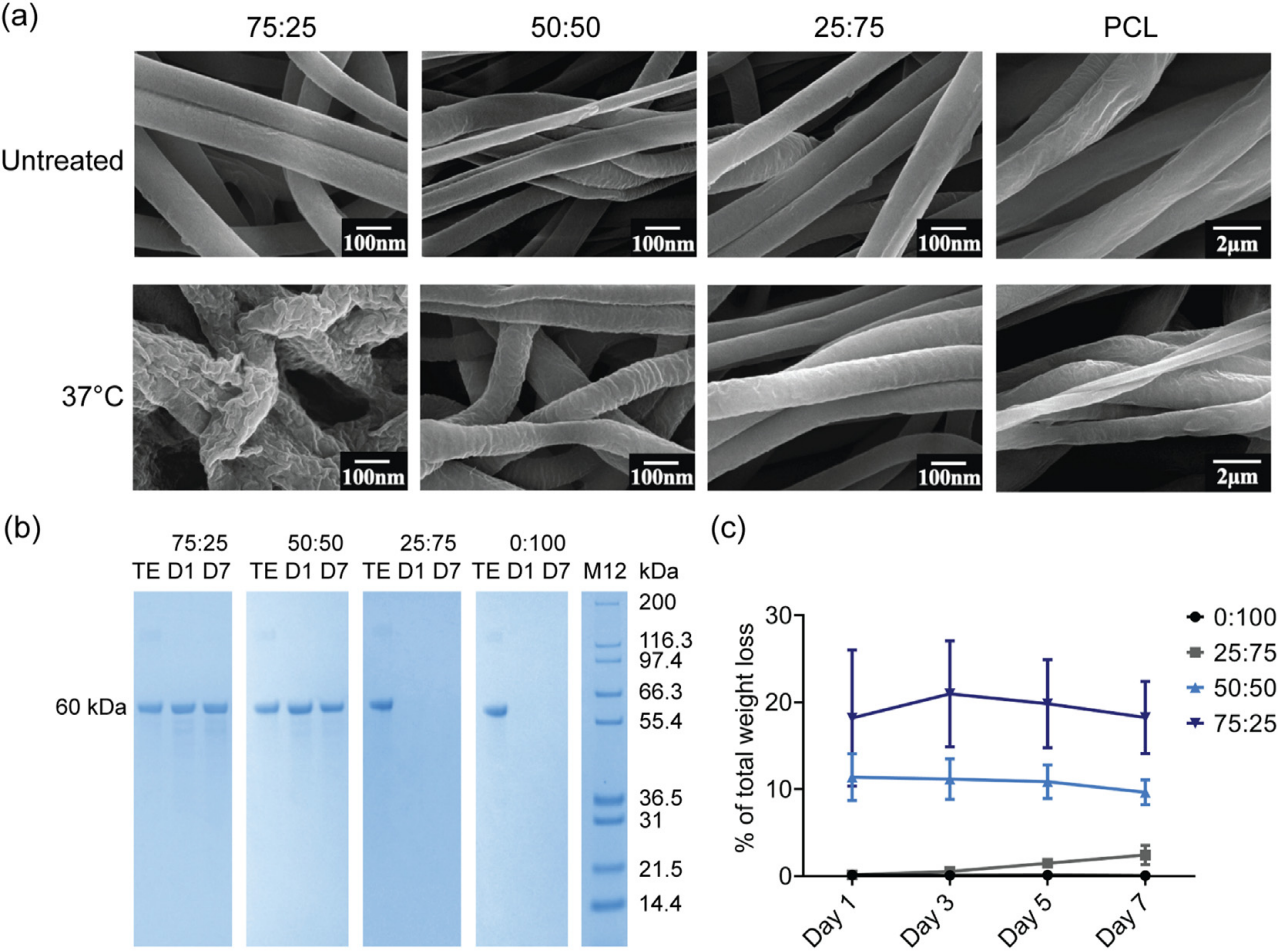
Effect of immersion under aqueous conditions on yarns. (a) SEM micrographs of tropoelastin:PCL electrospun yarns before and after water treatment for 24 h at 37 °C. (b) SDS-PAGE analysis of protein released from tropoelastin:PCL electrospun yarns after ethanol treatment and incubation in PBS at 37 °C for 1 (D1) or 7 (D7) days. TE - tropoelastin monomer, M12 - Mark12 protein standards. (c) Quantitative analysis of protein released from tropoelastin:PCL electrospun yarns after ethanol treatment and incubation in PBS at 37 °C for 1, 3, 5 and 7 days. Data are expressed as percent of total yarn mass (2 mg) and show the mean ± standard deviation, for each group n = 3.

SEM and protein loss analysis were used to assess the structural integrity and compositional stability of the yarns in aqueous solutions. Nanoscale SEM images indicated the effect on fibrous structures of immersing yarns in water at 37 °C for 24 h (Fig. 3a). Structural change was most evident for the 75:25 yarn. Prior to immersion, all yarns comprised separately formed fibers with smooth surfaces. Following immersion, the 75:25 nanofibers partially fused and displayed rough wrinkled surfaces, whereas other yarns maintained their distinct fibrous structures with decreasing levels of surface coarseness evident in the 50:50 and 25:75 yarns. No obvious change in surface smoothness was apparent for the 0:100 yarns. As PCL degrades slowly under aqueous conditions, we attributed the cause of the relatively rapid observed structural changes to tropoelastin leaching; this was confirmed by SDS-PAGE (Fig. 3b) and NanoDrop (Fig. 3c) analysis. SDS-PAGE analysis revealed leached tropoelastin monomer that was derived from 75:25 and 50:50 yarns within 24 h of immersion in PBS at 37 °C. NanoDrop measurements indicated that this initial burst release of tropoelastin resulted in an approximate loss in total yarn weight of 18 ± 8% of 75:25 and 11 ± 3% of 50:50 within the first 24 h. No further significant loss was evident by the seven-day mark. Tropoelastin was released from the 25:75 formulation over 7 days, resulting in a 2 ± 1% loss in total weight. We emphasize that tropoelastin leaching from the yarns was intentional, as the protein is soluble in aqueous solutions and, in order to ensure the biological benefits of tropoelastin, we did not wish to use cross-linking agent to stabilize it. Although cross-linking would have retained tropoelastin within the yarns for a longer period of time [50], the yarns were designed to deliver an initial release of tropoelastin, to promote elastogenesis by fibroblasts [51] and angiogenesis [52,53]. As a result, exposure to an aqueous environment promoted dissolution of surface tropoelastin for the higher ratio yarns, while we retained the remaining tropoelastin within the yarns to provide a prolonged release triggered by PCL degradation. On this basis, we propose that these versatile biomaterials can be tuned to degrade at tailored rates.

**Fig. S2:**
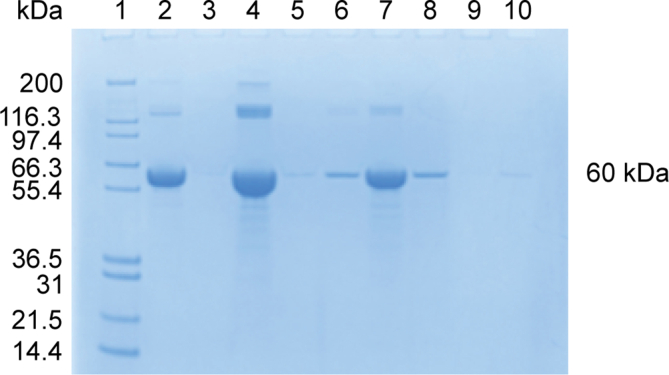
SDS-PAGE analysis of tropoelastin leaching following sterilization of 50:50 tropoelastin:PCL meshes. Gel lanes contain Mark 12 Standards (lane 1), tropoelastin (5 μg; lane 2); tropoelastin leached from a 50:50 tropoelastin:PCL mesh after immersion for 30 min in 100% ethanol (lane 3) followed by immersion in PBS for a further 1 h (lane 4), 3 h (lane 5), 18 h (lane 6) and ethylene oxide-treated 50:50 tropoelastin:PCL mesh after immersion for 30 min in PBS (lane 7) followed by immersion in PBS for a further 1 h (lane 8), 3 h (lane 9), 18 h (lane 10). Comparison of leached tropoelastin bands to the intensity of the control tropoelastin band indicate ~11% initial weight loss following ethanol treatment and PBS immersion and ~14% initial weight loss following ethylene oxide sterilization and PBS immersion.

As the 50:50 yarns were produced in multimeter lengths, showed superior structural integrity in aqueous conditions and generated a potentially pro-healing burst release of tropoelastin, this tropoelastin:PCL blend was chosen for surgical mesh fabrication and further characterization. An open weave mesh was constructed to allow the tropoelastin to function in two ways: (1) upon implantation, its availability on the surface of the mesh allowed for the aforementioned initial burst release of the protein, in order to promote tissue integration and early cell growth; (2) as the PCL portion of the mesh would breakdown over time, the tropoelastin embedded in the yarns would be slowly released. To ensure sterility without affecting this burst, we confirmed that ethylene oxide sterilization of 50:50 meshes did not prevent the initial release of tropoelastin from the mesh (Supp. Fig. S2).

### 3.5 Mechanical characterization

**Fig. 4:**
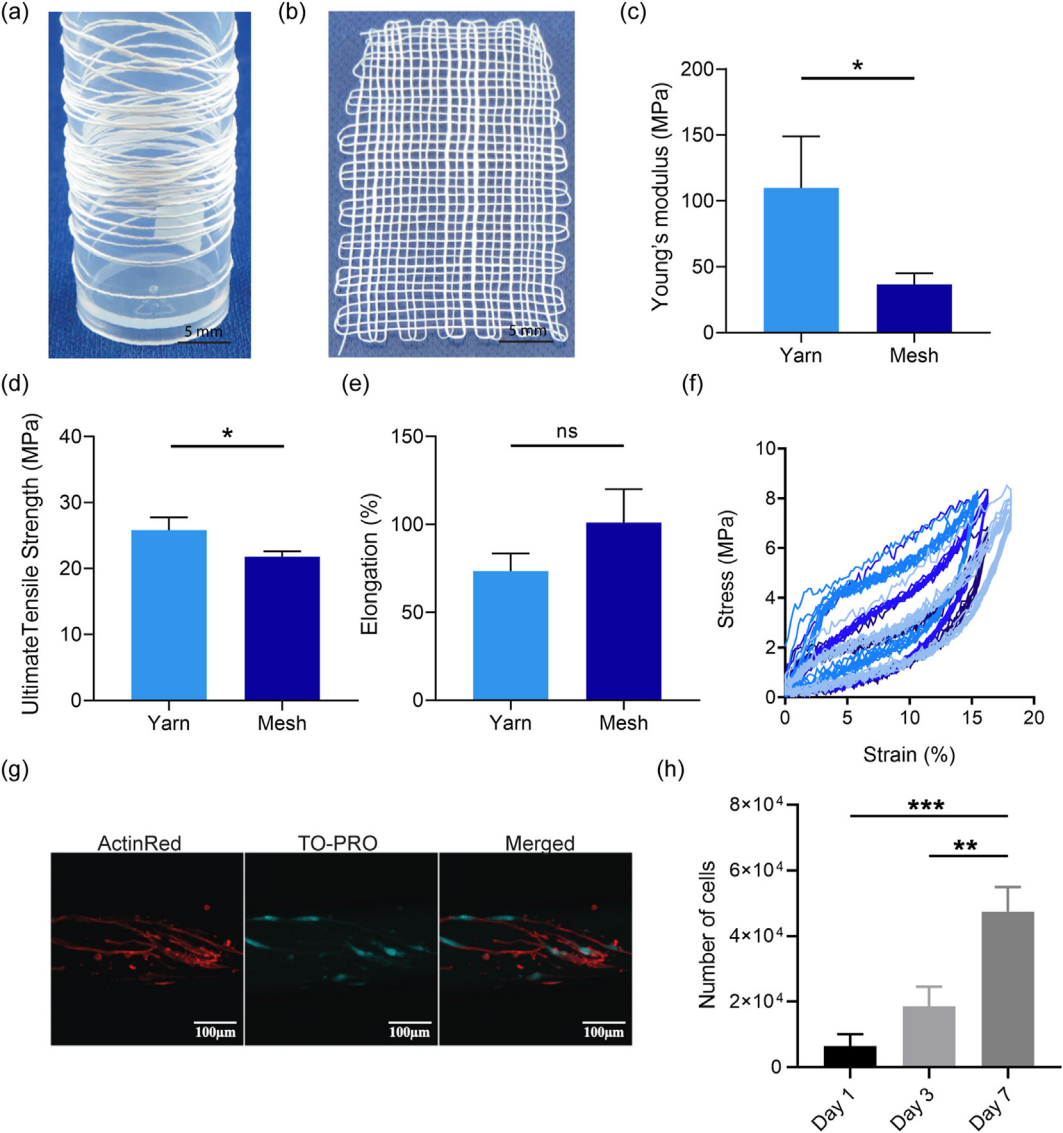
50:50 tropoelastin:PCL yarn and mesh. Capacity for electrospinning of (a) long continuous strands of 50:50 yarns allows for (b) woven mesh fabrication. Young's modulus (c), ultimate tensile strength (d) and elongation at break (e) of 50:50 yarns and meshes hydrated in PBS at 37 °C. Cyclic tensile testing (f) of 50:50 meshes indicating hysteresis and non-permanent deformation (n = 4). (g) Confocal images of human dermal fibroblasts cultured on 50:50 yarns after 7 days incubation in cell culture media at 37 °C. Cells were stained with ActinRed (red) to view F-Actin, and TO-PRO 3 iodide (cyan) to image nuclei, followed by merged images. (h) Fibroblast proliferation on 50:50 meshes over seven days (n = 3). (For interpretation of the references to colour in this figure legend, the reader is referred to the Web version of this article.)

The mechanical properties of 50:50 yarn (Fig. 4a) and mesh woven from a 50:50 yarn (Fig. 4b) were compared. Yarns and meshes were equilibrated in PBS at 37 °C for 20 h prior to testing, measurements were then performed in a PBS bath at 37 °C. The yarn displayed a higher Young's modulus (Fig. 4c) and ultimate tensile strength (UTS; Fig. 4d), 109.8 ± 39.0 MPa and 25.8 ± 1.9 MPa respectively, than were seen for the mesh, 36.5 ± 8.5 MPa and 21.8 ± 0.8 MPa respectively. While we cannot exclude the possibility that there was insufficient loading that had been made beforehand to allow for all the fibers to collectively contribute and respond to the load, the meshes went through cyclic stretching prior to the measurement, so it is more likely that the decreased Young's modulus for the mesh was a consequence of the free movement of the warp over the weft. The mesh modulus is close to the modulus of 34.3 ± 13.0 MPa reported for nulliparous ovine vaginal tissue, but double that reported for parous sheep, which is more similar to the 6–12 MPa reported for women who had experienced menopause before and after [54,55]. The percent elongation at break (Fig. 4e) was similar for both yarn and mesh, 73.5 ± 10% and 101 ± 19% respectively.

Consistent with the hysteresis of tropoelastin-silk meshes and tropoelastin-based sponges [24,56], tropoelastin:PCL meshes displayed hysteresis of 49.1 ± 7.7% under cyclic tensile testing, where the meshes recovered after each cycle and displayed stable behavior. The curves for all cycles overlaid, except for the first cycle (Fig. 4f). This indicated their suitability for implantation with non-permanent deformation under comparable strain conditions.

### 3.6 Cell culture

For a biomaterial to be successful, it must be able to support cellular growth. In this study, human dermal fibroblasts were cultured on 50:50 tropoelastin:PCL yarns and meshes for 7 days. Fibroblasts provided a model system that reflected the known contribution of fibroblasts following POP surgery and subsequent repair [57]. Confocal images (Fig. 4g) of the yarns indicated that cells were able to populate their surface. Proliferation studies (Fig. 4h) demonstrated a 7.4-fold increase in cell numbers from 24 h to 7 days after seeding where the number of cells increased 7.4-fold from 6.4 × 10^3^ ± 3.7 × 10^3^ on day 1–47.5 × 10^3^ ± 7.4 × 10^3^ on day 7 of culture.

### 3.7 Biomaterial integration in sheep vagina

**Fig. 5:**
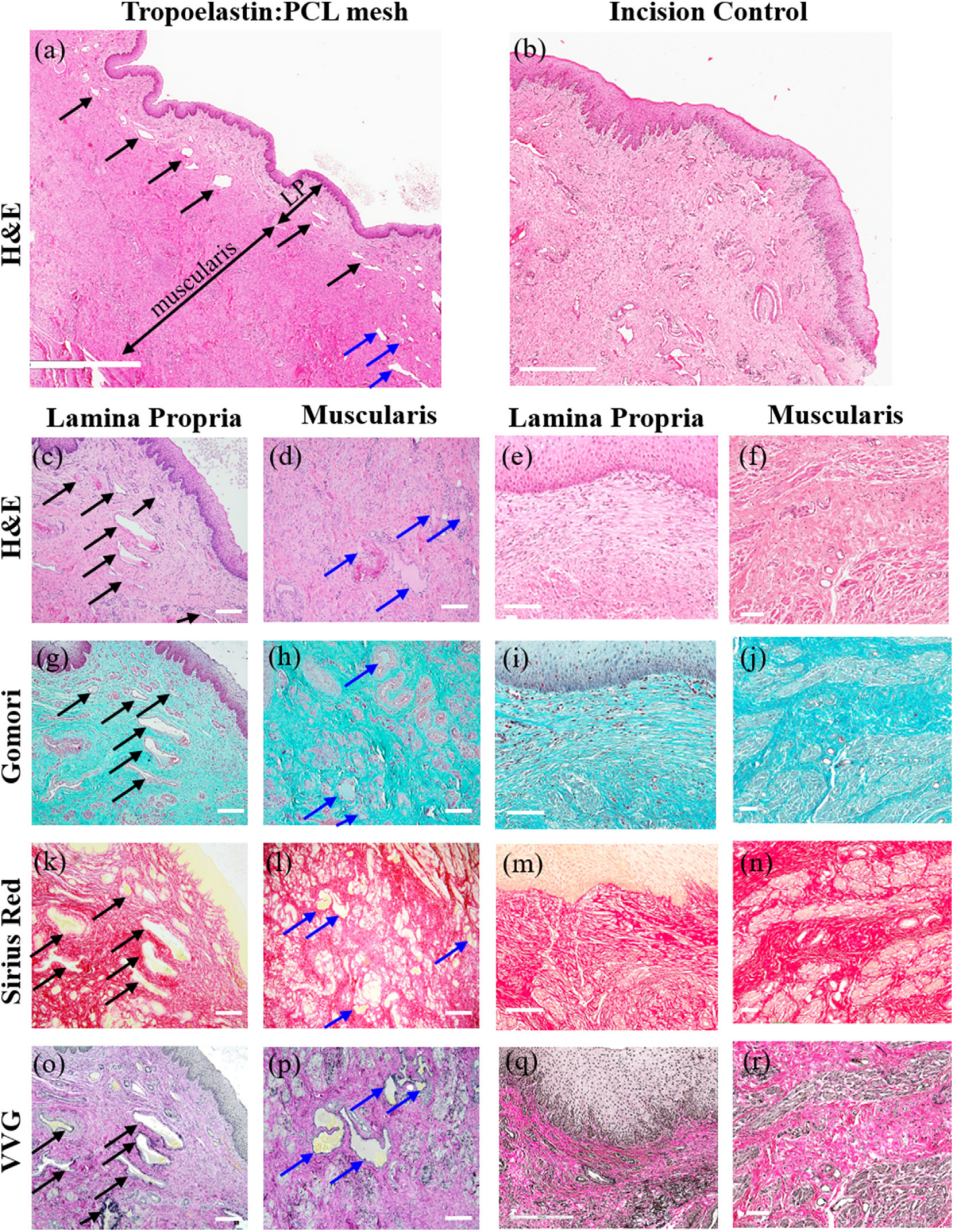
Histology of tropoelastin:PCL mesh after 4 weeks implantation in the ovine vagina. H&E (a) showing panoramic view of 50:50 tropoelastin:PCL mesh mainly between the lamina propria and muscularis (black arrows) and in the muscularis (blue arrows), compared with (b) incision control. Higher power images show the (c, d) mesh and (e, f) incision control, respectively. Collagen staining by Gomori (blue) (g–j) and Sirius Red (red) (k–n) show collagen around mesh filaments (arrows) and in ECM. VVG staining (o–r) show a few black elastin fibers in the tissue and around the tropoelastin of the mesh filament surface (arrows). LP, lamina propria. Representative images n = 1 each of mesh implanted and incision control ewes. Scale bars: (a–b) 2 mm, (c–r) 200 μm. (For interpretation of the references to colour in this figure legend, the reader is referred to the Web version of this article.)

**Fig. 6:**
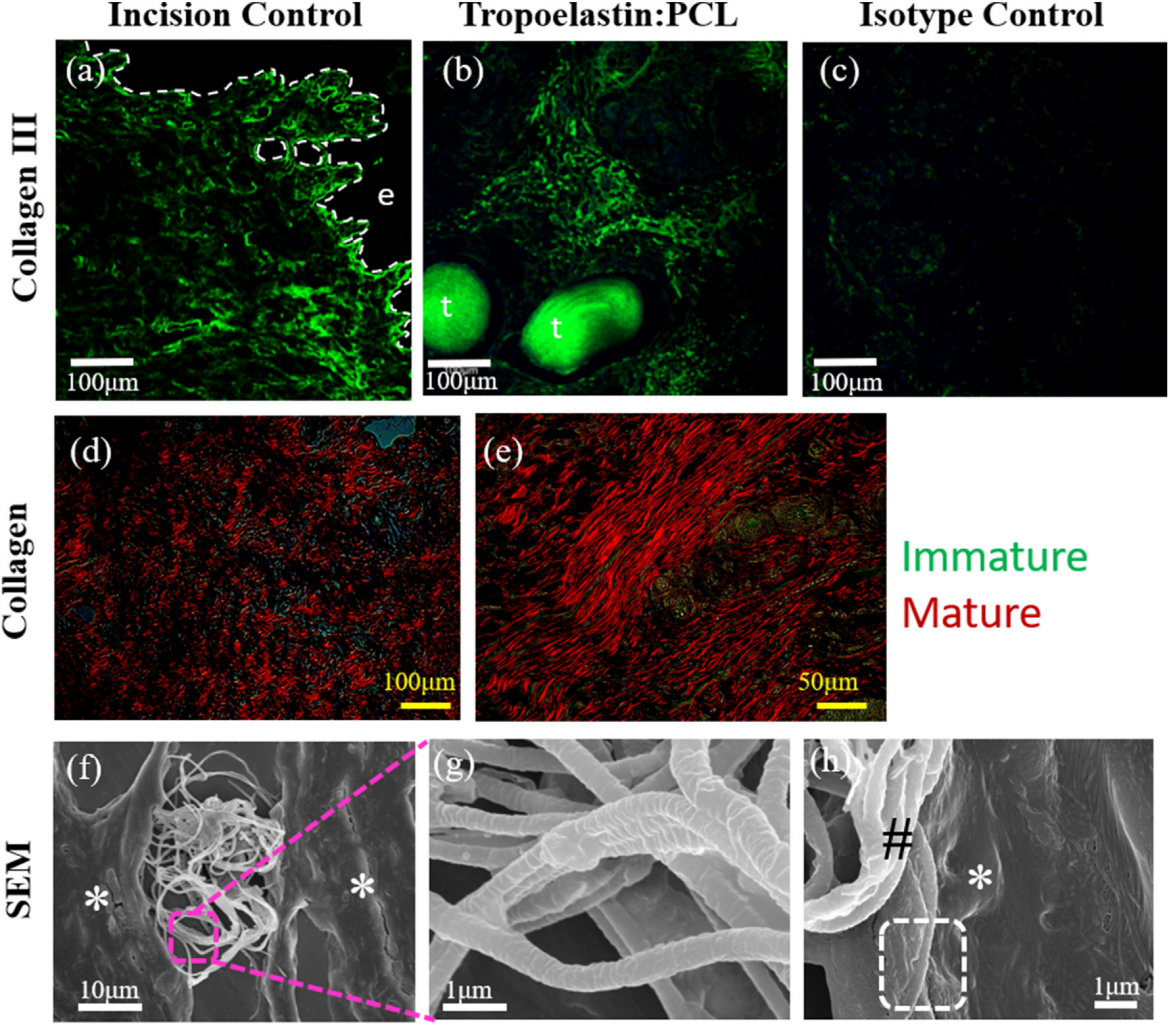
Immunofluorescence images showing deposited collagen III (green) in explanted ovine vaginal tissue after 30 days (a) near the incision site and (b) around filaments of the tropoelastin:PCL mesh, in contrast to the (c) isotype control. (d, e) Birefringence of Sirius Red-stained vaginal tissue shows mature (red) and immature collagen (green) after 30 days of implanted tropoelastin:PCL mesh in ovine vagina. SEM images of explanted ovine vaginal tissue with (f) tropoelastin:PCL mesh show (g) integrity of the yarn structure and (h) integration (white dotted box) of mesh (#) with host tissue (∗) after 30 days. Dotted line indicates epithelial lamina propria border. Key: e, epithelium; t, tropoelastin:PCL. (For interpretation of the references to colour in this figure legend, the reader is referred to the Web version of this article.)

Lack of mesh integration leading to mesh erosion/exposure is one of the major causes of complications associated with commercial polypropylene (PP) meshes [9,58]. PP meshes disrupt the vaginal ECM and lead to an undesired immune response [9] underlying the importance of evaluating novel meshes that better integrate with the vaginal tissue [58]. We have previously undertaken a comparative study with PP mesh and newly designed knitted meshes in rats and showed improved host responses, new collagen deposition and greater neovascularization with the new mesh designs [59]. Preclinical studies using degradable nanofiber meshes have shown no erosion and similar integration into ovine vaginal tissue [15], indicating their potential as alternative meshes. Our research has shown that sheep vagina closely resembles the human vagina [60], making it an appropriate cost-effective large animal model for POP. As a result, the ovine model has been widely used to evaluate vaginal mesh integration and performance [15,43,61]. A panoramic image shows the 50:50 tropoelastin:PCL mesh was inserted between the lamina propria and muscularis of the ovine vaginal wall (Fig 5a, c) although some filaments were also in the muscularis (Fig. 5d) after 30 days. Comparison with the incision control (Fig. 5b, e, f), revealed that tropoelastin:PCL performed well, in that it was associated with little disruption to the architecture of the ovine vagina after 30 days in both lamina propria and muscularis. Three connective tissue stains verified the lack of tissue architecture disruption, unlike PP meshes [62] with no scar type collagen evident in Gomori's (Fig. 5g and h) and Picrosirius Red stained sections from the explanted tissue (Fig. 5k and l). The collagen component of both the lamina propria and muscularis were similar to the incision control (Fig. 5i, j, m, n) and healthy multiparous ewes as we have previously reported [42]. In healing tissue, newly synthesized (immature) collagen is characterized by deposition of type III collagen, which raises the immature type III to mature type I collagen ratio, as a new ECM matrix is generated to provide support for cells. With the VVG elastin stain, it was apparent that the tropoelastin component was still present after 30 days as it reacted with the stain (Fig. 5o and p). The incision control showed deposition of elastin fibers in the lamina propria around the incision site (Fig. 5q), confirming the capacity of the injured vagina to synthesizes new elastin fibers. Collagen III was detected in the incision (control) (Fig. 6a) and around the tropoelastin:PCL filaments (Fig. 6b). Birefringence of Sirius Red-stained tissues revealed small amounts of immature collagen (green) deposition amongst mature (red) collagen (Fig. 6d and e) in similar to proportions to healthy multiparous ovine vagina [42]. In addition to this hallmark of tissue integration of biomaterial in the host tissue, scanning electron micrographs showed integration of the mesh with the host tissue (Fig. 6f–h), unlike PP meshes which lead to tissue disruption [62]. High resolution SEM imaging further confirmed that tropoelastin:PCL maintained structural integrity after 30 days (Fig. 6g). Collectively these results show thorough integration of tropoelastin:PCL in sheep vaginal tissue after 30 days. This is in contrast to various stiff products such as Gynemesh or Avaulta, discontinued PP meshes used in transvaginal surgery which severely disrupted the muscularis of vagina in sheep [62] and non-human primates [63].

**Fig. 7:**
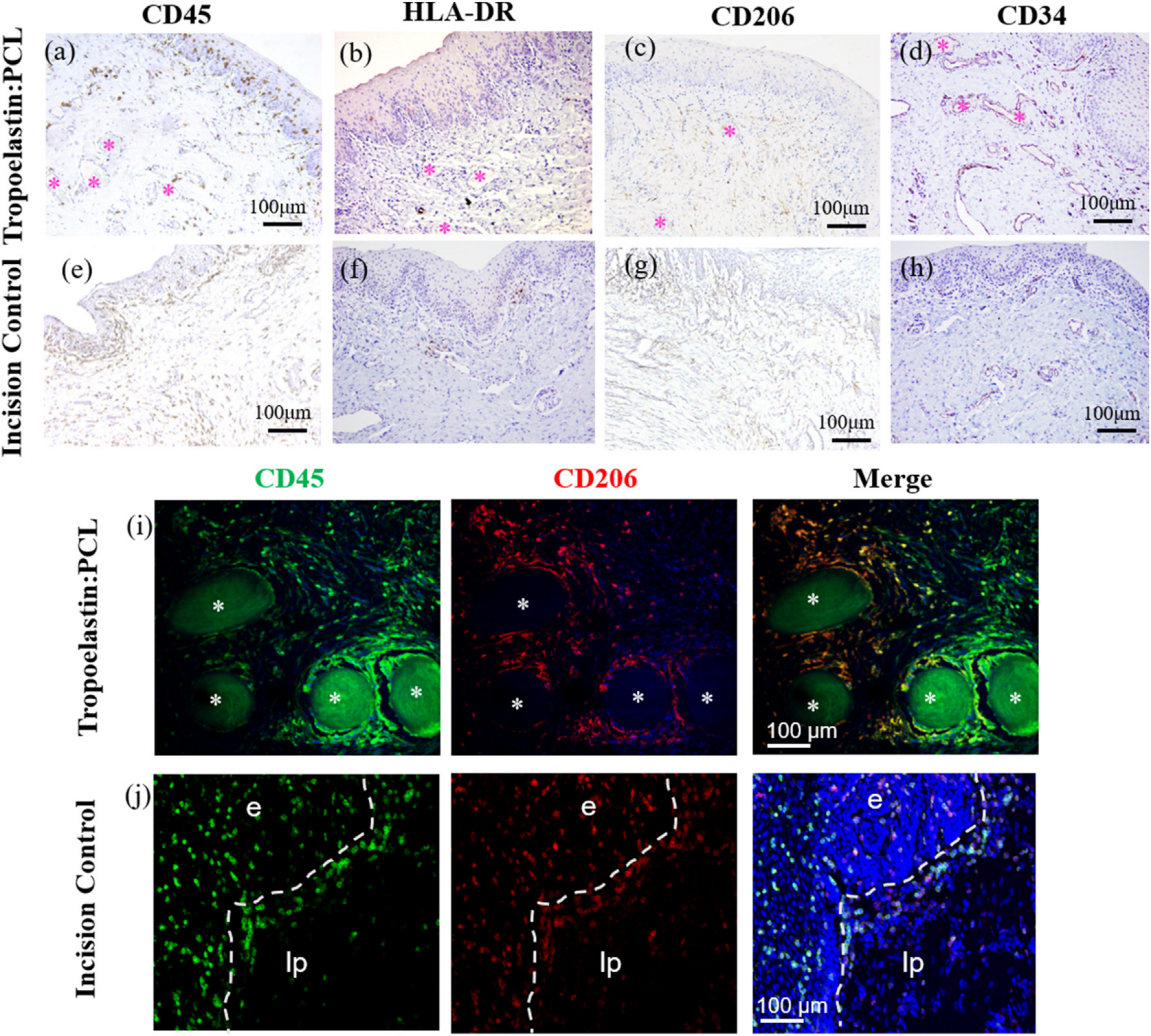
Analysis of foreign body response to tropoelastin:PCL mesh implanted in an ovine vaginal surgery model of POP. Immunohistochemistry for (a) CD45 (b) HLA-DR (c) CD206 and (d) CD34 stained positive cells in brown in the epithelium and lamina propria of a tropoelastin:PCL explant, in comparison to (e–h) incision control ovine vaginal tissues. Immunofluorescence shows colocalization of CD45^+^ leukocytes (green) and CD206^+^ M2 macrophages (red; merge = yellow) at the (i) tropoelastin:PCL filament tissue interface. In tissue more distant to the filaments, CD45^+^ leukocytes (green in merge panel) were either M1 inflammatory or M0 uncommitted macrophages. (j) CD45^+^ leukocytes (green) colocalize with CD206^+^ M2 macrophages (red) in incision control ovine vaginal tissue. Representative images of n = 1 mesh implanted and n = 1 incision control ewe. (For interpretation of the references to colour in this figure legend, the reader is referred to the Web version of this article.).

Another important aspect of mesh biocompatibility *in vivo* is the degree of foreign body response elicited by implanted biomaterials [12,20]. Macrophage-mediated foreign body responses to meshes are critical in determining the fate of implanted biomaterials [9]. We found CD45^+^ leukocytes distributed in the tissue around the tropoelastin:PCL mesh (Fig. 7a) that was similar to the incision control (Fig. 7e). While there are limited antibodies that reliably detect antigens in ovine tissues, human leukocyte antigen DR isotype (HLA-DR) has recently emerged as a potential M1 marker (M1 indicates proinflammatory macrophages) [15]. On this basis, very few M1 macrophages were observed in both the tropoelastin:PCL implanted and incision control vagina (Fig. 7b, f), consistent with a low pro-inflammatory response to this mesh. Wound healing M2 macrophages were also observed in tropoelastin:PCL-treated explanted vagina (Fig. 7c) and the incision control (Fig. 7g). Angiogenesis that follows *in vivo* mesh integration is a key sign of successful integration in the host. Consistent with this integration, we observed the presence of blood vessels in ovine tissues in the vicinity of our implant, as evidenced by CD34^+^ cells lining blood vessel profiles. This appeared higher than in incision control vaginal tissues (Fig. 7d, h). The promotion of neovascularization in ovine tissues (Fig. 7d), in addition to an anti-inflammatory immune response, are each consistent with successful integration of our tropoelastin:PCL in ovine vagina. Colocalization studies showed that a substantial proportion of CD45^+^ leukocytes were CD206^+^ M2 macrophages, particularly at the tropoelastin:PCL mesh tissue interface (Fig. 7i). Those CD45^+^ leukocytes not immunostained with CD206 (Fig. 7i) may have been M0 macrophages but unlikely to be M1 macrophages, based on M1 marker, HLA-DR, immunohistochemical staining (Fig. 7b). In summary, for vaginal applications, it is desirable that meshes degrade slowly over time as mechanical reinforcement of the pelvic organ support structures is essential for POP repair [13,14,23]. The slow degradation rate of tropoelastin:PCL helps to avoid rapid material degradation. For these reasons, tropoelastin:PCL mesh shows potential as a suitable implant biomaterial as demonstrated by proof-of-concept results in our ovine POP model.

Expanded studies will allow for quantification of the immune response and other parameters indicating tissue integration accompanied by statistical analysis and biomechanical evaluation [62]. These studies should assess effects beyond 30 days, to a minimum of 90 days and preferably 180 days, in order to fully judge the potential of the tropoelastin:PCL for vaginal repair surgery and ensure long term integration, assess tissue ingrowth, chronic inflammation and material degradation as well as adverse outcomes such as excessive fibrosis, exposure or erosion [61,62].

A relevant incision control of ovine vaginal tissue was included for comparison of histological data. The present study qualitatively shows, in a highly relevant animal model of transvaginal surgery used ahead of clinical testing, that tropoelastin:PCL is a promising material for further development as a vaginal mesh for treating women with POP. Indeed, a much larger, adequately powered study that includes a comparator, clinically available mesh and healthy tissue control is planned in the near future.

## 4 Conclusions

In an ovine model of POP, transvaginal insertion of the 50:50 tropoelastin:PCL mesh demonstrated complete integration into the host vaginal tissue after 30 days *in vivo* and importantly showed no erosion. Commensurate with *de novo* supporting tissue synthesis required to take over the mechanical role of the mesh as it resorbs, 50:50 tropoelastin:PCL mesh implantation resulted in new collagen deposition into the tissue around the mesh and maintained structural integrity. These features will provide mechanical reinforcement of the weakened vaginal wall thereby enabling provision of long-term support of pelvic organs. On this basis, we propose these meshes as a new class of alternative, degradable pelvic organ prolapse mesh products.

## Author contributions

BA, KL, SMM, ZW, SM and SD contributed to experimentation made before clinical testing, tissue analysis, data interpretation and manuscript writing.

CEG and ASW contributed to conceptual framework, experimental design, interpretation of results and manuscript writing.

## Declaration of competing interest

The authors declare the following financial interests/personal relationships which may be considered as potential competing interests: ASW is the Scientific Founder of Elastagen Pty Ltd, now sold to Allergan and Abbvie.

## References

[1] Jelovsek J.E. (2007). Lancet.

[2] Dwyer L., Kearney R. (2018). Obstet. Gynaecol. Reprod. Med..

[3] DeLancey J.O. (2005). Am. J. Obstet. Gynecol..

[4] Ganj F.A. (2009). Int. UrogynEcol. J. Pelvic Floor Dysfunct..

[5] Silva W.A., Karram M.M. (2005). Curr. Opin. Obstet. Gynecol..

[6] Friedman T. (2018). International Urogynecology Journal.

[7] Smith F.J. (2010). Obstet. Gynecol..

[8] Liang R. (2013). BJOG An Int. J. Obstet. Gynaecol..

[9] Nolfi A.L. (2016). Am. J. Obstet. Gynecol..

[10] O'Brien F.J. (2011). Mater. Today.

[11] Freed L.E. (1994). Nature.

[12] Paul K. (2019). Acta Biomater..

[13] Mukherjee S. (2019). Biomacromolecules.

[14] Vashaghian M. (2018). Neurourol. Urodyn..

[15] Hympánová L. (2020). Eur Urol Focus.

[16] Mukherjee S. (2020). Front. Pharmacol..

[17] Paul K. (2020). Nanomaterials.

[18] Ulery B.D. (2011). J. Polym. Sci. B Polym. Phys..

[19] Liu H. (2006). Int. J. Nanomed..

[20] Mukherjee S. (2019). Interface Focus.

[21] Hutmacher D.W. (2000). Biomaterials.

[22] Wu S. (2017). Acta Biomater..

[23] Gargett C.E. (2019). Curr. Opin. Urol..

[24] Aghaei-Ghareh-Bolagh B. (2019). Acta Biomater..

[25] Xue J. (2019). Chem. Rev..

[26] Ali U. (2012). J. Textil. Inst..

[27] Moutos F.T., Guilak F. (2008). Biorheology.

[28] Rodgers U.R., Weiss A.S. (2005). Pathol. Biol..

[29] Shen N., Anand Ramamurthi C.K. (2016). Elastic Fiber Matrices.

[30] Wise S.G. (2014). Acta Biomater..

[31] Bax D.V. (2009). J. Biol. Chem..

[32] Jin E. (2016). Regenerative Engineering and Translational Medicine.

[33] Yeo G.C. (2015). Advanced Healthcare Materials.

[34] Li M. (2005). Biomaterials.

[35] Rnjak-Kovacina J. (2011). Biomaterials.

[36] Liu H. (2014). Biomaterials.

[37] Díaz E. (2014). J. Nanomater..

[38] Ghosal K. (2017). AAPS PharmSciTech.

[39] Bölgen N. (2005). J. Biomater. Sci. Polym. Ed..

[40] Zhang Y.Z. (2005). Biomacromolecules.

[41] Mukherjee S. (2011). Adv. Funct. Mater..

[42] Emmerson S. (2017). Sci. Rep..

[43] Emmerson S. (2019). Biomaterials.

[44] Young N. (2017). Int Urogynecol J.

[45] McKenna K.A. (2012). Acta Biomater..

[46] Wise S.G. (2011). Acta Biomater..

[47] Chen M. (2007). Tissue Eng..

[48] Kim G.-M. (2013). J. Mater. Sci. Mater. Med..

[49] Haris P.I., Severcan F. (1999). J. Mol. Catal. B Enzym..

[50] Nivison-Smith L. (2010). Acta Biomater..

[51] Mithieux S.M., Weiss A.S. (2017). Acta Biomater..

[52] Wang Y. (2015). Advanced Healthcare Materials.

[53] Mithieux S.M. (2018). Advanced Healthcare Materials.

[54] Knight K.M. (2016). International Urogynecology Journal.

[55] Baah-Dwomoh A. (2016). Appl. Mech. Rev..

[56] Wang R. (2019). Biomaterials.

[57] Su K. (2014). Acta Biomater..

[58] Liang R. (2013). BJOG.

[59] Ulrich D. (2012). PloS One.

[60] Ulrich D. (2014). PloS One.

[61] Darzi S. (2016). Acta Biomater..

[62] Feola A. (2015). Am. J. Obstet. Gynecol..

[63] Shaffer R.M. (2019). Am. J. Obstet. Gynecol..

